# Predictive value of ^99m^Tc-MAA-based dosimetry in personalized ^90^Y-SIRT planning for liver malignancies

**DOI:** 10.1186/s13550-023-01011-3

**Published:** 2023-07-03

**Authors:** Mercedes Riveira-Martin, Azadeh Akhavanallaf, Zahra Mansouri, Nicola Bianchetto Wolf, Yazdan Salimi, Alexis Ricoeur, Ismini Mainta, Valentina Garibotto, Antonio López Medina, Habib Zaidi

**Affiliations:** 1 Genetic Oncology, Radiobiology and Radiointeraction Research Group, Galicia Sur Health Research Institute, Vigo, Spain; 2 Division of Nuclear Medicine and Molecular Imaging, Diagnostic Department, Geneva University Hospital, 1211 Geneva, Switzerland; 3 Service of Radiology, Geneva University Hospital, 1211 Geneva, Switzerland; 4 Centre for Biomedical Imaging (CIBM), Geneva, Switzerland; 5 Geneva Neuroscience Centre, Geneva University, Geneva, Switzerland; 6 Department of Medical Physics and RP, Hospital do Meixoeiro (GALARIA), Vigo, Spain; 7 Department of Nuclear Medicine and Molecular Imaging, University Medical Centre Groningen, University of Groningen, Groningen, Netherlands; 8 Department of Nuclear Medicine, University of Southern Denmark, Odense, Denmark; 9 Department of Radiology, Rehabilitation and Physiotherapy, Medicine School, Complutense University of Madrid, Madrid, Spain

**Keywords:** Selective internal radiation therapy, ^90^Y radioembolization, Dosimetry, SPECT, Theragnostics

## Abstract

**Background:**

Selective internal radiation therapy with ^90^Y radioembolization aims to selectively irradiate liver tumours by administering radioactive microspheres under the theragnostic assumption that the pre-therapy injection of ^99m^Tc labelled macroaggregated albumin (^99m^Tc-MAA) provides an estimation of the ^90^Y microspheres biodistribution, which is not always the case. Due to the growing interest in theragnostic dosimetry for personalized radionuclide therapy, a robust relationship between the delivered and pre-treatment radiation absorbed doses is required. In this work, we aim to investigate the predictive value of absorbed dose metrics calculated from ^99m^Tc-MAA (simulation) compared to those obtained from ^90^Y post-therapy SPECT/CT.

**Results:**

A total of 79 patients were analysed. Pre- and post-therapy 3D-voxel dosimetry was calculated on ^99m^Tc-MAA and ^90^Y SPECT/CT, respectively, based on Local Deposition Method. Mean absorbed dose, tumour-to-normal ratio, and absorbed dose distribution in terms of dose-volume histogram (DVH) metrics were obtained and compared for each volume of interest (VOI). Mann–Whitney U-test and Pearson’s correlation coefficient were used to assess the correlation between both methods. The effect of the tumoral liver volume on the absorbed dose metrics was also investigated. Strong correlation was found between simulation and therapy mean absorbed doses for all VOIs, although simulation tended to overestimate tumour absorbed doses by 26%. DVH metrics showed good correlation too, but significant differences were found for several metrics, mostly on non-tumoral liver. It was observed that the tumoral liver volume does not significantly affect the differences between simulation and therapy absorbed dose metrics.

**Conclusion:**

This study supports the strong correlation between absorbed dose metrics from simulation and therapy dosimetry based on ^90^Y SPECT/CT, highlighting the predictive ability of ^99m^Tc-MAA, not only in terms of mean absorbed dose but also of the dose distribution.

**Supplementary Information:**

The online version contains supplementary material available at 10.1186/s13550-023-01011-3.

## Background

Liver cancer is currently the third leading cause of cancer death and the sixth most commonly diagnosed cancer worldwide [1]. Selective internal radiation therapy (SIRT) or Radioembolization (RE) with Yttrium-90 (^90^Y) labelled glass or resin microspheres is a widely used technique to treat primary and secondary liver malignancies, such as hepatocellular carcinoma (HCC), intrahepatic cholangiocarcinoma (ICC) and metastatic cancer spread to the liver, the first type being the most treated with this technique. SIRT aims to selectively irradiate liver tumours by administering radioactive microspheres through hepatic arteries, while sparing healthy tissue. Several studies demonstrated that the tumour absorbed dose is highly correlated with treatment response and tumour control probability, whereas non-tumoral liver absorbed dose correlates with induced toxicity and with radioembolization-induced liver disease (REILD) [2–6]. Therefore, an accurate treatment planning is required to ensure the safety and efficacy of the therapy by evaluation of the delivered dose to tumoral and non-tumoral liver tissue as well as extra-hepatic regions [7, 8]. Treatment planning based on patient-specific dosimetry is predicted to significantly improve clinical efficacy and cost-effectiveness and is therefore expected to be used in future trials of targeted internal radiation therapy [9, 10].

^90^Y-SIRT procedure applies a theragnostic concept for therapy planning and verification using similar radiopharmaceutical pairs. In the first step, the treatment is simulated using ^99m^Tc-macroaggregated albumin (^99m^Tc-MAA), as a surrogate for ^90^Y microspheres. Following the infusion of the ^99m^Tc-MAA, the patient undergoes planar scintigraphy and Single-Photon Emission Computed Tomography/Computed Tomography (SPECT/CT), which allows the visualization of any extrahepatic distribution (in particular the lung localization caused by a high liver/lung shunt and the quantitative estimation of lung shunt fraction, LSF), the radiopharmaceutical biodistribution and pre-therapy dosimetry [8, 11] and furthermore guides for patient stratification and therapy optimization. In the second step, post-therapy imaging is performed to verify the distribution of the delivered dose from the ^90^Y-microsphere infusion, which can be performed using either a Bremsstrahlung SPECT/CT (bSPECT) or Positron Emission Tomography/Computed Tomography (PET/CT) [12].

The simulation technique is based on the hypothesis that the biodistribution of ^99m^Tc-MAA and ^90^Y-microspheres is identical, due to the relatively similar size and density of the microspheres [13, 14]. However, studies indicated large variations in correlations between activity distribution of ^99m^Tc-MAA and ^90^Y microspheres [15], ranging from good to poor correlations (mostly in tumoral tissue) [16–25]. The bremsstrahlung X-ray spectrum makes quantitative bSPECT imaging challenging, and ^90^Y PET suffers from high bias and variability because of the limited positron emission (32 per million decays). However, the latter has proven to be qualitatively and quantitatively superior when the radioactivity is highly concentrated, as is the case in RE [26–28]. Nevertheless, not only ^90^Y bSPECT is more affordable and widespread worldwide [29], but also PET/CT scanners recommended for this purpose are those having time-of-flight (TOF) capability, which are even less available to most centres than SPECT/CT [30, 31]. Therefore, personalized dosimetry studies in ^90^Y-SIRT based on bSPECT are still relevant and necessary [24].

The most basic dosimetry methods, such as the MIRD mono-compartment method [32], base the activity calculation on the desired mean absorbed dose to the target liver, independent of tumour burden, assuming a homogeneous absorbed dose distribution over the target tumour. A more personalized approach is the partition model (PM), which evaluates the activity on three compartments with different uptakes (tumours, non-tumoral tissue and lungs) [14], maximizing the absorbed dose to the tumour while limiting cytotoxic dose to healthy tissue. However, these methods do not account for the heterogeneity of the absorbed dose deposition. On the contrary, dosimetry at the voxel level (voxel-based dosimetry) accounts for the non-uniformity of activity distribution. This technique allows to compute dose volume histograms (DVHs) as in external beam radiation therapy (EBRT) [33], potentially providing useful knowledge on dose–effect relationships [12]. However, unlike EBRT, voxel-based dosimetry in RE relies on nuclear medicine images, with poorer image quality, making it difficult to directly quantify dose effects [12]. Thus, although there are recent studies confirming the validity of voxel dosimetry in SIRT [34, 35], some studies raised doubts on its real benefits of in this area [31, 36].

There is a growing interest in personalized radionuclide therapy with an increasing number of clinical trials striving to unify protocols and mitigating uncertainties, as the positive impact of a personalized regimen compared to the standard model in ^90^Y-SIRT has been demonstrated [10] (response rate: 79% vs. 43%, respectively). In this context, a robust relationship between the pre-therapy absorbed dose estimation and therapeutic delivered dose is required to be broadly investigated and further established [11]. Hence, this study aims to investigate the correlation between pre-therapy ^99m^Tc-MAA SPECT/CT and post-therapy ^90^Y bSPECT/CT in a large cohort of patients, to try to mitigate uncertainty about the validity of ^99m^Tc-MAA simulation in a voxel level dosimetry.

## Methods

### Patients

This retrospective study included a cohort of 90 patients treated by SIRT at the Geneva University Hospital (Geneva, Switzerland) from January 2011 to December 2021 with glass microspheres. Patients received a pre-treatment administration of ^99m^Tc-MAA and a single treatment session with ^90^Y-microspheres. The study protocol was approved by the institution’s ethics committee, and all patients gave written informed content.

### Radioembolization and imaging

SIRT was performed according to the general procedure described in the literature [37]. All patients were treated with glass microspheres (Therasphere™; Boston Scientific, Marlborough, Massachusetts). Simulation was performed (mean ± standard deviation, SD) 21 ± 7 days before treatment with ^90^Y, according to the manufacturer’s guidelines, with an intra-arterial injection of 154 ± 13 MBq of ^99m^Tc-MAA. After simulation, planar and SPECT/CT imaging was performed to determine the extrahepatic shunts, in particular LSF, and confirm tumour coverage. Patients were excluded from therapy if the absorbed dose to the lungs was expected to exceed 30 Gy per treatment and/or 50 Gy in cumulative dose for those patients treated in several sessions. SPECT/CT images for simulation were obtained on a Symbia-T series camera (Siemens Healthcare, Erlangen, Germany) using low-energy high-resolution collimators, an energy window of 140 keV (15% energy window width) with 64 projections over a 180° angle (20–25 s per projection) within a 128 × 128 matrix. Tomograms were mostly reconstructed using a three-dimensional ordered-subset expectation maximization (OSEM3D) algorithm with 4 iterations and 8 subsets, including attenuation correction and a Gaussian filter of 5 mm. The ^90^Y treatment activity was determined based on the single-compartment partition model. Once the activity is defined, following the same procedure as during the simulation with ^99m^Tc-MAA, after catheterization of the hepatic artery by the interventional radiologist, the nuclear medicine physician administers the glass microspheres (activity of 2.6 ± 1.2 GBq) using the TheraSphere™ Yttrium-90 glass microspheres delivery system. The residual activity left in the vial is measured with an activity meter. After treatment, the patient undergoes ^90^Y bSPECT/CT imaging to ensure the proper distribution of the microspheres. The images were performed on the same scanner as the simulation but with an energy window centred in 90 keV (30% window width), 150 keV (60% window width) and 170 (50% window width) for 9, 65 and 16 patients, respectively. For most patients, 64 projections were acquired (60 projections for 12 patients and 32 for 3 patients) over a 180° angle (15–30 s per projection) within a 128 × 128 matrix. The reconstruction was performed using OSEM3D algorithm with 4 iterations and 8 subsets, including attenuation correction and a Gaussian filter of 5 mm.

### Workflow

#### Image segmentation

Three contours are performed according to the anatomical images: lesions, targeted lobe, and whole liver. Lesion and lobar segmentations are manually performed by an experienced nuclear medicine physician. The lesions were contoured on the diagnostic images on the baseline contrast-enhanced CT or magnetic resonance (MR), and the treated lobe on the attenuation-corrected CT (AC-CT) from the ^99m^Tc-MAA simulation. As recommended [38], we avoided using threshold-based tumour segmentation on SPECT images as it often does not represent true anatomical extent due to heterogeneous microsphere distribution. In large lesions with a visible necrotic core in CT, the core was excluded from the lesion volume [8]. Small tumours with a volume under 4 ml, considered equivalent of a 2-cm-diameter spherical lesions, were excluded from the analysis, as recommended [31, 39]. Whole-liver segmentation was automatically performed by a commercially available Artificial Intelligence Software (Limbus AI Inc., Canada, v1.6.0) on the AC-CT from the ^99m^Tc-MAA simulation.

#### Image registration

Hybrid images (SPECT/CT) are acquired under the same system matrix, thus are aligned by default. Simulation (^99m^Tc-MAA SPECT/CT) and therapy (^90^Y bSPECT/CT) images are manually co-registered to each other based on the anatomical information from their AC-CTs with a rigid transformation. The targeted lobe and whole-liver contours may help the user to align them in this step. Secondly, the diagnostic image (CT or MR) is co-registered to the simulation and therapy AC-CTs using a rigid transformation, in order to localize the lesions on SPECT/CTs. In both registrations, rigid transformation is used since the deformable one may not always correctly handle the differences in matrix and voxel size [40]. The diagnostic image, the multimodal SPECT/CT from therapy, the SPECT/CT image from simulation and all the segments (lesions, lobe and whole-liver) were resampled to the AC-CT from the ^99m^Tc-MAA simulation. All pre-processing was performed within 3D Slicer (v4.11.2).

#### Absorbed dose calculation

3D-voxel dosimetry was performed assuming the following statements: (1) there is permanent trapping of the microspheres (no biological clearance), (2) there is no activity shunt outside the liver, (3) there is no energy cross-talk among voxels by using the local energy deposition method, (4) the half-life ($${T}_{1/2})$$ of ^90^Y is 64.1 h, mean energy 0.93 MeV and the density of the liver is 1.05 g/cm^3^. The dosimetry calculations were performed using an in-house developed MATLAB code (MATLAB (2021a), Natick, Massachusetts: The MathWorks Inc) based on the calculations described by Moran et al. [41]. A detailed description of the pipeline is provided in the Additional file 1.

**Fig. 1:**
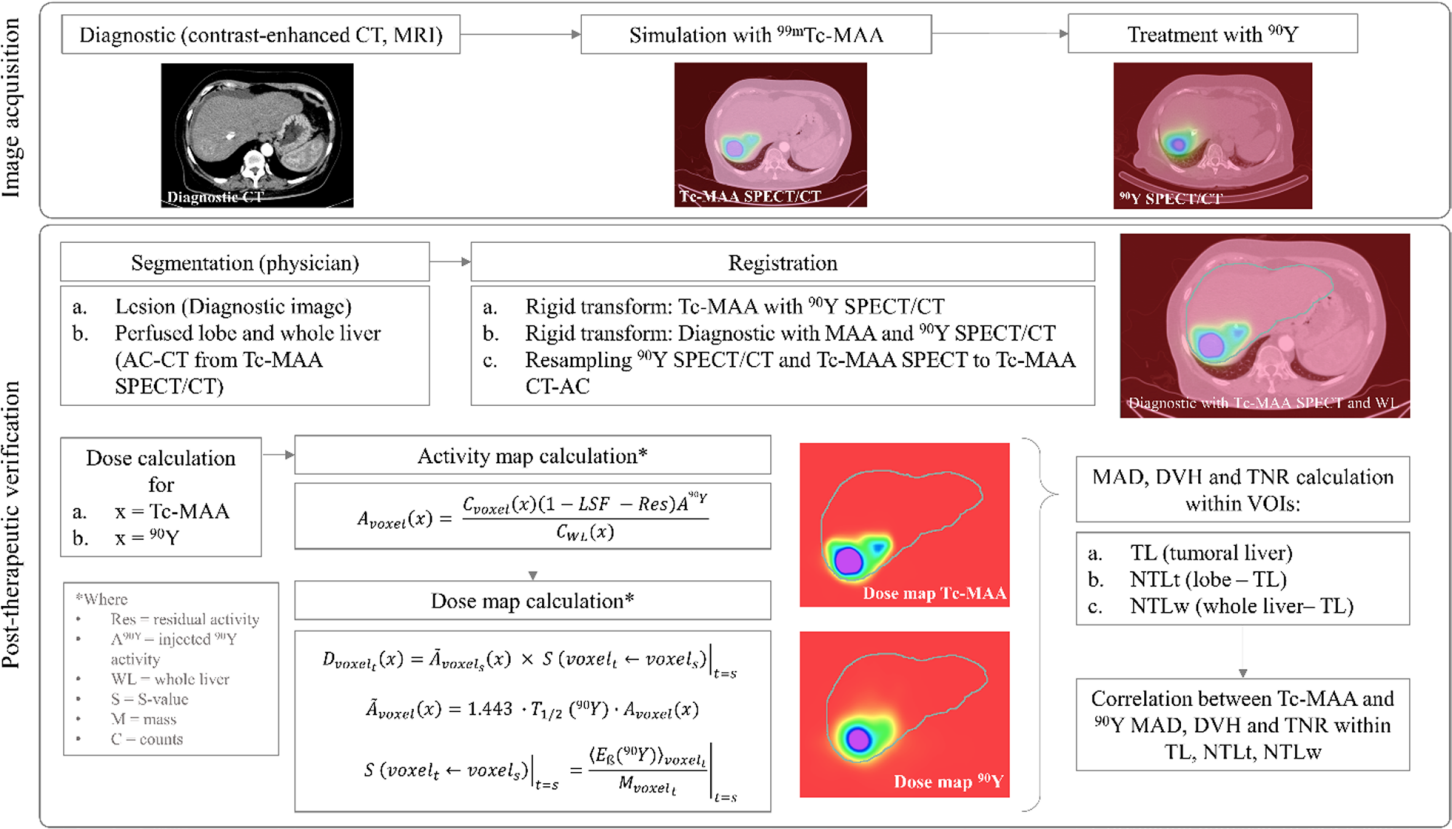
Followed workflow. The acquired images are the pre-treatment diagnostic CT or MR and the SPECT and AC-CT from both simulation and therapy. For the post-therapeutic verification, segmentation and registration is performed. From these images, we proceed to calculate the activity map and then, the dose map based on self-calibration strategy and the local energy deposition approach. Finally, the MAD, DVH and TNR are calculated within the three VOIs

We defined three volumes of interest (VOIs) to perform the dosimetry: Tumoral liver (TL), which is the sum of all the contoured lesions, and non-tumoral liver (NTL), discretizing this one into non-tumoral liver target (NTLt), which is the healthy tissue within the targeted lobe, and non-tumoral whole liver (NTLw), which is the healthy tissue within the whole liver. From the obtained dose maps, the mean absorbed dose (MAD) and the DVH curves were calculated for each VOI. From DVHs, we calculated the following absorbed dose metrics: D50, D70, D95, V120 and V205 for TL; and D50, D70 and D95, V20, V50, V90 for NTL (Dx: minimum dose received by x% of the volume; Vx: the percentage of the volume receiving at least x Gy). In addition, V205 was evaluated based on the recommended minimum dose-cut-off for therapy response in the literature, while V50 and V90 due to the dose limits recommended for the normal liver [33]. Finally, the tumour-to-normal liver ratio (TNR) was calculated for each patient for both simulation and therapy with respect NTLt (TNR-NTLt) and NTLw (TNR-NTLw) (i.e., considering the activities from ^99m^Tc-MAA and ^90^Y SPECTs and the mass from NTLt and NTLw). In this equation, A_TL_ and A_NTL_ is the activity obtained from the TL and NTL VOIs, respectively, and M_TL_ and M_NTL_ is the mass of the corresponding VOIs. The followed workflow is summarized in Fig. 1.

1 $$\mathrm{TNR}= \frac{{A}_{\mathrm{TL}}/{M}_{\mathrm{TL}}}{{A}_{\mathrm{NTL}}/{M}_{\mathrm{NTL}}}$$

### Statistical analysis

Absorbed dose metrics (MAD and DVH metrics) were obtained for each VOI (TL, NTLt, NTLw) for both simulation (^99m^Tc-MAA) and therapy (^90^Y) and normalized by ^90^Y injected activity. Considering all patients, the mean ± SD from each metric was calculated. Values of TNRs (TNR-NTLt, TNR-NTLw) were also calculated for each patient and averaged over all patients.

Metrics based on simulation ^99m^Tc-MAA SPECT and therapy ^90^Y SPECT were compared using the Wilcoxon’s rank sum test (Mann–Whitney U-test), assuming 95% significance level, thus considering statistically significant differences between simulation and therapy metrics if *p*-value (*P*) was less than 0.05. Pearson’s correlation coefficient (*r*) was used to assess the degree of linear correlation between both metrics, considering: 0 < *r* < 0.3 very weak; 0.3 < *r* < 0.5 weak; 0.5 < *r* < 0.7 moderate; 0.7 < *r* < 0.9 strong; and 0.9 < *r* < 1.0 very strong correlation [25]. A linear regression model was implemented to correlate the ^90^Y delivered dose with pre-therapy ^99m^Tc-MAA images. The mean relative difference was also calculated for all metrics. The Kruskal–Wallis test was used to evaluate the potential effect of tumoral volume and the difference between absorbed dose metrics with respect to demographic variables. The statistical analysis was performed in Python 3.9.

## Results

### Study population

**Fig. 2:**
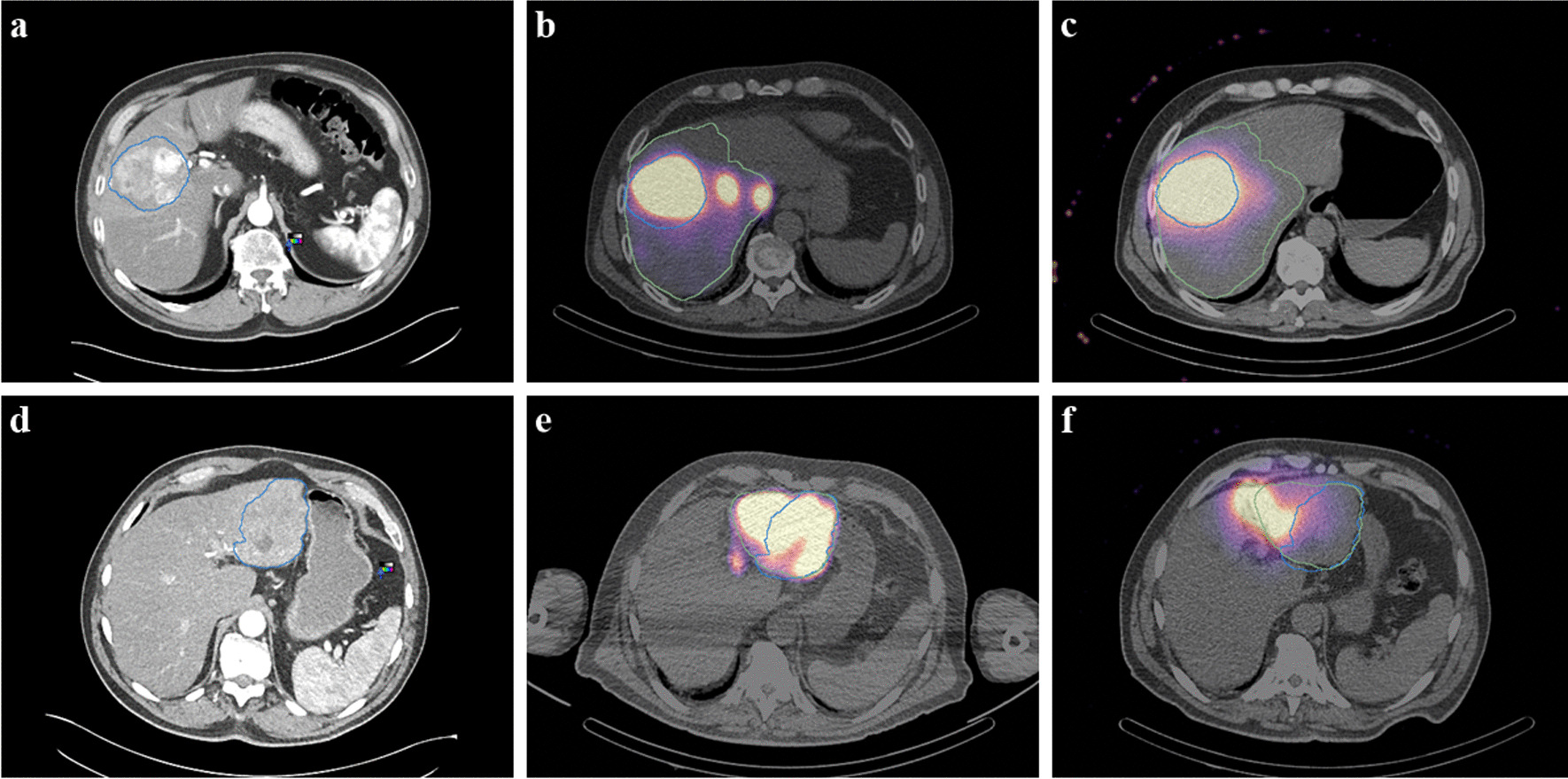
An example of patient images showing agreement (top: **a**, **b**, **c**) and other example showing disagreement (bottom: **d**, **e**, **f**) between simulation SPECT with 99mTc-MAA and therapy bSPECT with 90Y. **a**, **d** Diagnostic contrast-enhanced CT. **b**, **e** SPECT/CT images of the pre-treatment simulation with 99mTc-MAA. **c**, **f** SPECT/CT images of the therapy session with 90Y. The segments corresponding to the perfused lobe and to the tumour are depicted in green and blue, respectively. Both examples correspond to HCC patients

**Fig. 3:**
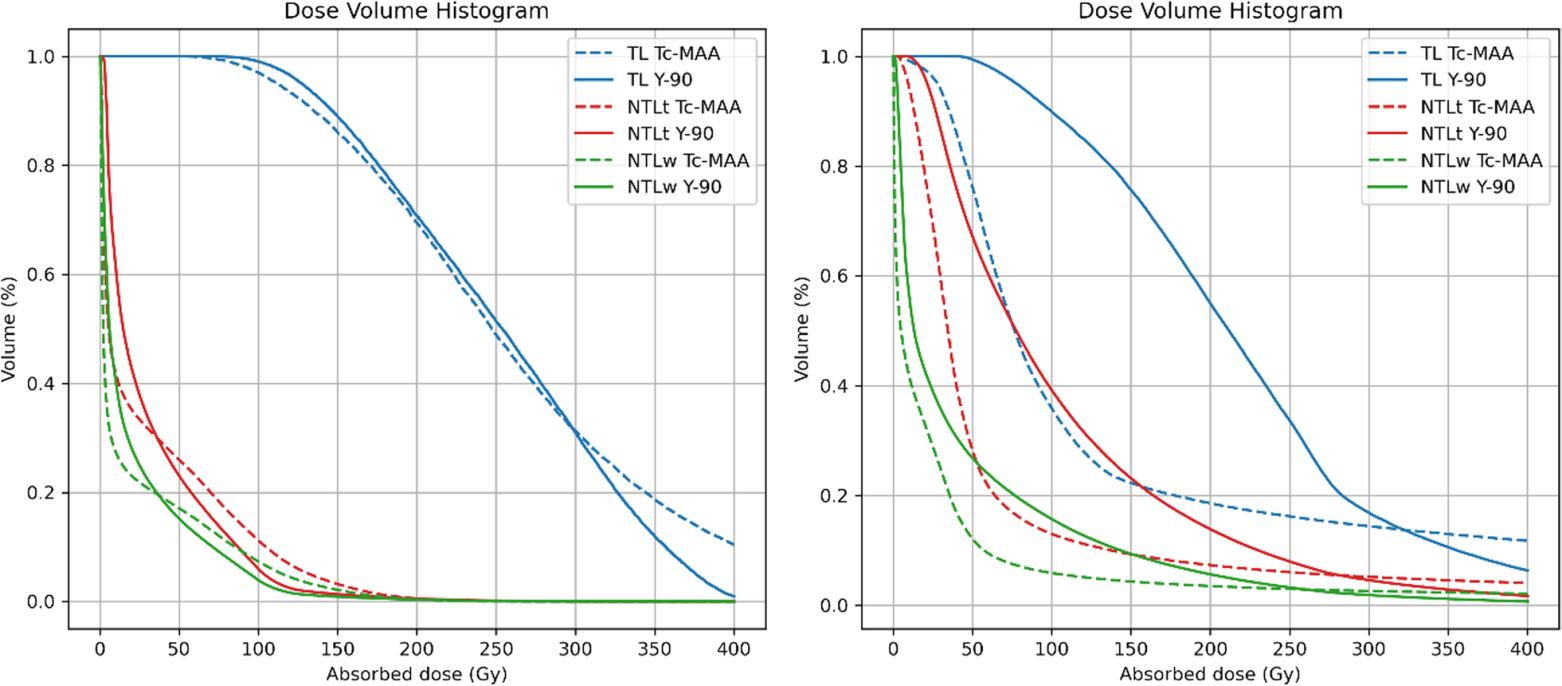
Dose-volume histograms of a patient with visual match from the SPECT (left) and mismatch (right) for all VOIs (TL, NTLt, NTLw), both patients of HCC with a single lesion

Of the 90 patients included in the study, 11 were excluded because the simulation and therapy activity maps did not visually match, in order to minimize the bias that physical differences between simulation and therapy can cause, such as differences in catheter positioning. An example of a patient showing visual agreement and disagreement is shown in Fig. 2, respectively, and example of the DVH curves in Fig. 3. Therefore, a total of 79 patients were analysed. Among these patients, 71 were treated of HCC, 4 of ICC and 4 of metastatic colorectal cancer (mCRC). A total of 98 lesions were analysed, 88 HCC, 4 ICC and 6 mCRC. Demographic information is summarized in Table 1.

**Table 1:** Baseline demographics and clinical characteristics

Patient characteristics	
Sex (M/F)	14 (17.7%) / 65 (82.3%)
Age (y)	69 [26–90]
Tumour type	
HCC	71 (89.8%)
ICC	4 (5.1%)
mCRC	4 (5.1%)
Normal liver and tumoral liver volumes	
TL (ml)	198.03 [4.56–2228.42]
NTLt (ml)	955.67 [13.46–2039.70]
NTLw (ml)	1562.25 [727.40–2980.48]
Lobe (left) (ml)	644.35 [209.93–1995.80]
Lobe (right) (ml)	1478.25 [484.50–2762.90]
Whole Liver (ml)	1845.09 [819.19–3458.50]
Tumour involvement^a^	
Total (%)	18.90 [0.50–95.10]
Patients with < 5%	14 (17.7%)
Patients with 5–10%	12 (15.1%)
Patients with 10–25%	28 (35.4%)
Patients with 25–50%	14 (17.7%)
Patients with > 50%	11 (13.9%)
Number of lesions	
Patients with 1 lesion	66 (77.2%)
Patients with 2 lesions	7 (8.8%)
Patients with 3 lesions	6 (13.9%)
Diagnostic image modality	
Contrast-enhanced CT	59 (74.7%)
Contrast-enhanced MRI	20 (25.3%)
Simulation (^99m^Tc-MAA) and therapy (^90^Y) characteristics	
Treated lobe (left/right)	19 (24.1%) / 60 (75.9%)
LSF (%)	8.0 [0.6–21.0]
Waiting period^b^ (d)	20 [9–47]
^99m^Tc-MAA activity (MBq)	152.0 [60.0–190.0]
^90^Y activity (GBq)	2.57 [0.74–5.9]
HCC (GBq)	2.50 [0.74–5.5]
ICC (GBq)	3.28 [2.2–4.2]
mCRC (GBq)	3.90 [1.9–5.9]

Results are presented as median [range] or n (%)

^a^Percentage of tumoral tissue volume to the lobe volume

^**b**^Waiting period stands for the days between simulation with ^99m^Tc-MAA and therapy with ^90^Y

### Absorbed dose metrics for TL, NTLt and NTLw

**Fig. 4:**
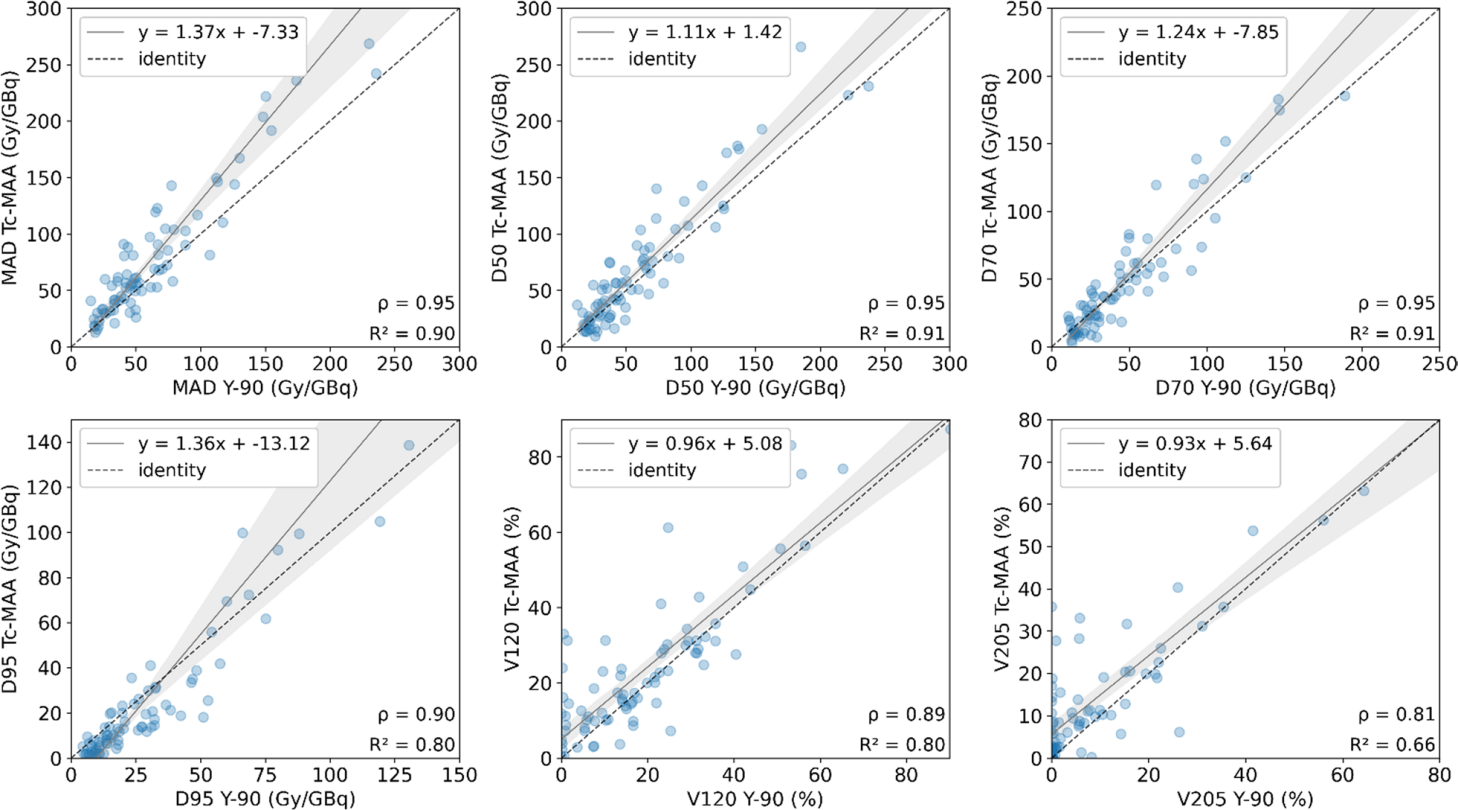
Correlations plots for different dosimetry metrics derived from TL: mean dose (MAD), D50 and D70 (top) and D95, V120 and V205 (bottom). For visualization purposes, in each graph the axes are set to equal length, leaving some points out of frame. The entire images are found on Additional file 1: Fig. S1

The normalized MAD for the total tumoral tissue (TL) is 85.36 ± 79.97 Gy/GBq and 67.55 ± 55.34 Gy/GBq calculated on ^99m^Tc-MAA and ^90^Y images, respectively﻿ (Table 2). There is a strong correlation between MAD, D50 and D70 (*r* = 0.95, *P* < 0.001) obtained from ^99m^Tc-MAA with respect to those derived from ^90^Y imaging, followed by D95 (*r* = 0.89, *P* < 0.001), V120 (*r* = 0.89, *P* < 0.001) and V205 (*r* = 0.81, *P* < 0.001). No statistically significant differences were found according to the Wilcoxon’s test, except for D95 and V205. Figure 4 shows the correlation plots and linear regression models all absorbed dose metrics.

**Table 2:** Absorbed dose distribution parameters (MAD, D50, D70, D95 (Gy/GBq) and V120, V205, V20, V50, V90 (%)) compared between simulation (^99m^Tc-MAA) and therapy (^90^Y) for all cases in TL, NTLt and NTLw

Segment	Metric	^99m^Tc-MAA	^90^Y	Wilcoxon *p*-value	Pearson's *r* correlation	Relative difference (%) mean [range]^a^
TL	MAD (Gy/GBq)	85.36 ± 79.97	67.55 ± 55.34	0.11	0.95 (*P* < .001)	25.64 [− 47.32–170.38]
	D50 (Gy/GBq)	71.57 ± 64.25	62.82 ± 54.53	0.52	0.95 (*P* < .001)	14.06 [− 63.17–203.4]
	D70 (Gy/GBq)	53.88 ± 58.27	49.8 ± 44.75	0.66	0.95 (*P* < .001)	1.53 [− 78.95–110.75]
	D95 (Gy/GBq)	27.46 ± 43.14	29.85 ± 28.44	0.01*	0.89 (*P* < .001)	− 26.44 [− 89.72–112.56]
	V120 (%)	25.41 ± 21.73	21.24 ± 20.29	0.15	0.89 (*P* < .001)	–
	V205 (%)	13.86 ± 17.21	8.87 ± 15.06	< 0.001*	0.81 (*P* < .001)	–
NTLt	MAD (Gy/GBq)	32.57 ± 20.37	32.28 ± 16.92	0.67	0.97 (*P* < .001)	− 2.36 [− 59.57–60.26]
	D50 (Gy/GBq)	22.67 ± 18.42	26.68 ± 16.22	0.01*	0.93 (*P* < .001)	− 20.38 [− 92.5–134.02]
	D70 (Gy/GBq)	13.5 ± 12.53	18.98 ± 12.43	< 0.001*	0.88 (*P* < .001)	− 34.26 [− 96.11–170.74]
	D95 (Gy/GBq)	4.04 ± 4.57	9.67 ± 6.85	< 0.001*	0.79 (*P* < .001)	− 62.13 [− 97.29–31.55]
	V20 (%)	32.8 ± 20.68	39.81 ± 21.88	< 0.001*	0.96 (*P* < .001)	− 18.71 [− 66.70–15.50]
	V50 (%)	21.71 ± 15.38	24.83 ± 16.61	0.04*	0.92 (*P* < .001)	− 9.41 [− 85.24–103.02]
	V90 (%)	12.38 ± 9.59	11.97 ± 8.37	0.96	0.88 (*P* < .001)	36.40 [− 93.10–1857.72]
NTLw	MAD (Gy/GBq)	18.67 ± 8.37	20.58 ± 7.46	0.10	0.95 (*P* < .001)	− 11.48 [− 60.30–17.82]
	D50 (Gy/GBq)	8.24 ± 7.05	13.83 ± 7.43	< 0.001*	0.84 (*P* < .001)	− 46.00 [− 92.31–53.27]
	D70 (Gy/GBq)	3.20 ± 4.20	7.98 ± 4.99	< 0.001*	0.82 (*P* < .001)	− 65.44 [− 95.21–5.99]
	D95 (Gy/GBq)	0.88 ± 1.72	3.1 ± 2.53	< 0.001*	0.82 (P < .001)	− 73.2 [− 96.66–35.30]
	V20 (%)	18.5 ± 8.06	24.86 ± 8.88	< 0.001*	0.88 (P < .001)	− 26.54 [− 73.53–17.12]
	V50 (%)	11.71 ± 6.15	13.78 ± 6.85	0.03*	0.84 (P < .001)	− 9.07 [− 85.78–345.82]
	V90 (%)	6.58 ± 3.87	6.78 ± 4.34	0.92	0.86 (P < .001)	115.36 [− 91.64–8574.56]

^*^Wilcoxon's *p*-value *P* < 0.05

^a^In cases where there was a null value in the quotient, the result is not shown (–)

**Fig. 5:**
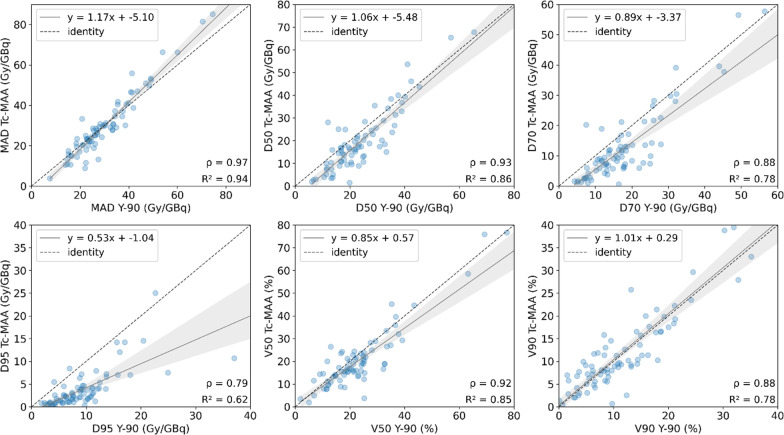
Correlations plots for different dosimetry metrics derived from NTLt: mean dose (MAD), D50 and D70 (top) and D95, V50 and V90 (bottom). For visualization purposes, the correlation for V20 is not shown, and in each graph the axes are set to equal length, leaving some points out of frame. The entire images are found on Additional file 1: Fig. S2

**Fig. 6:**
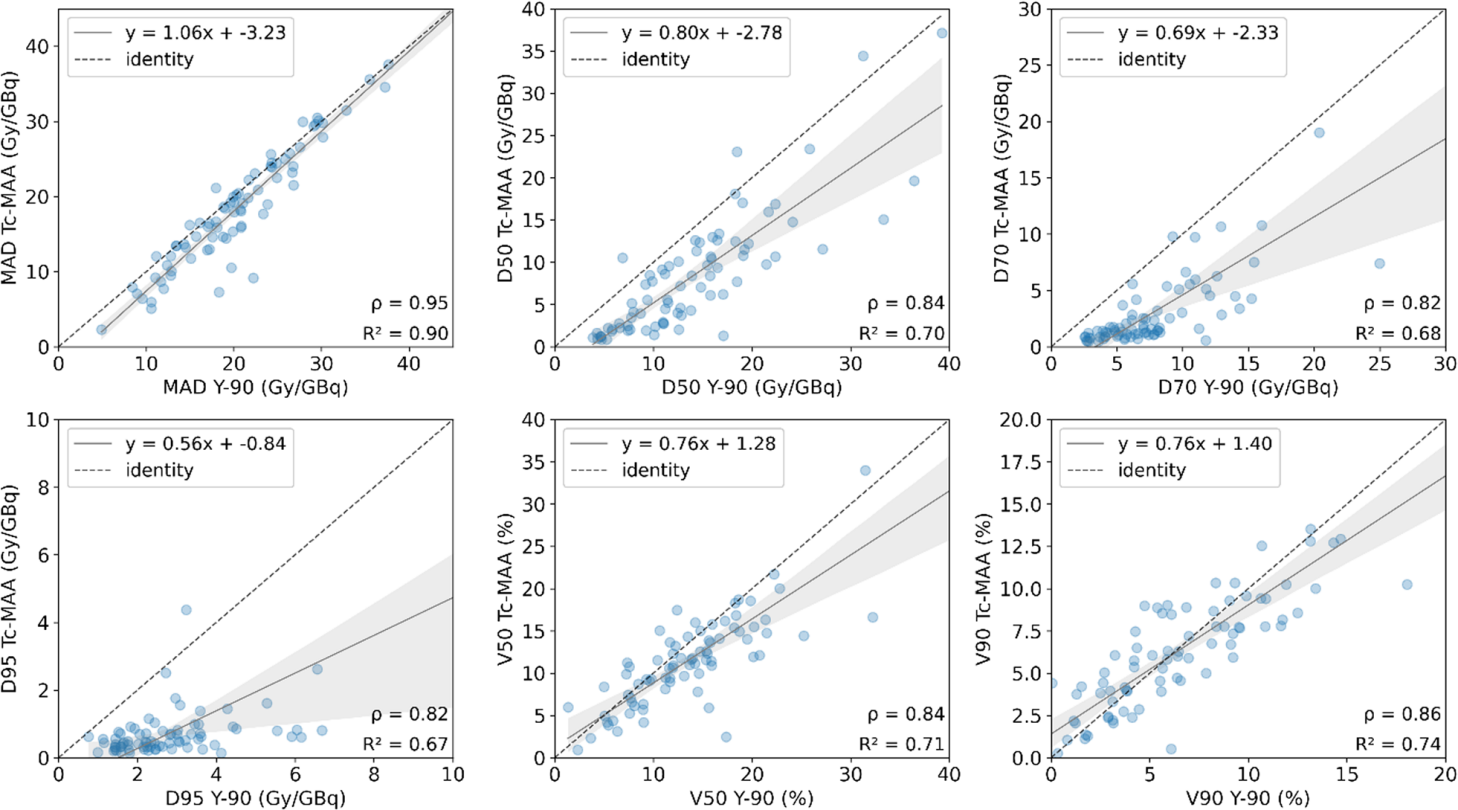
Correlations plots for different dosimetry metrics derived from NTLw: mean absorbed dose (MAD), D50 and D70 (top) and D95, V50 and V90 (bottom). For visualization purposes, the correlation for V20 is not shown, and in each graph the axes are set to equal length, leaving some points out of frame. The entire images are found on Additional file 1: Fig. S3

For NTLt, normalized MAD from simulation resulted in 32.57 ± 20.37 Gy/GBq, and 32.28 ± 16.92 Gy/GBq from therapy. A strong correlation was found for MAD (*r* = 0.97, *P* < 0.001), D50 (*r* = 0.93, *P* < 0.001), V20 (*r* = 0.96, *P* < 0.001) and V50 (*r* = 0.92, *P* < 0.001), followed by D70 (*r* = 0.88, *P* < 0.001), D95 (*r* = 0.79, *P* < 0.001) and V90 (*r* = 0.88, *P* < 0.001). For NTLw, the strongest correlation is found for MAD (*r* = 0.95, *P* < 0.001) and less than 0.90 for the rest of the parameters. However, for both NTLt and NTLw only MAD and V90 presented no significant differences between simulation and therapy (*P* > 0.05). Regression plots are represented in Figs. 5 and 6, respectively, for NTLt and NTLw. Results are summarized in Table 2.

### Tumoral volume

**Fig. 7:**
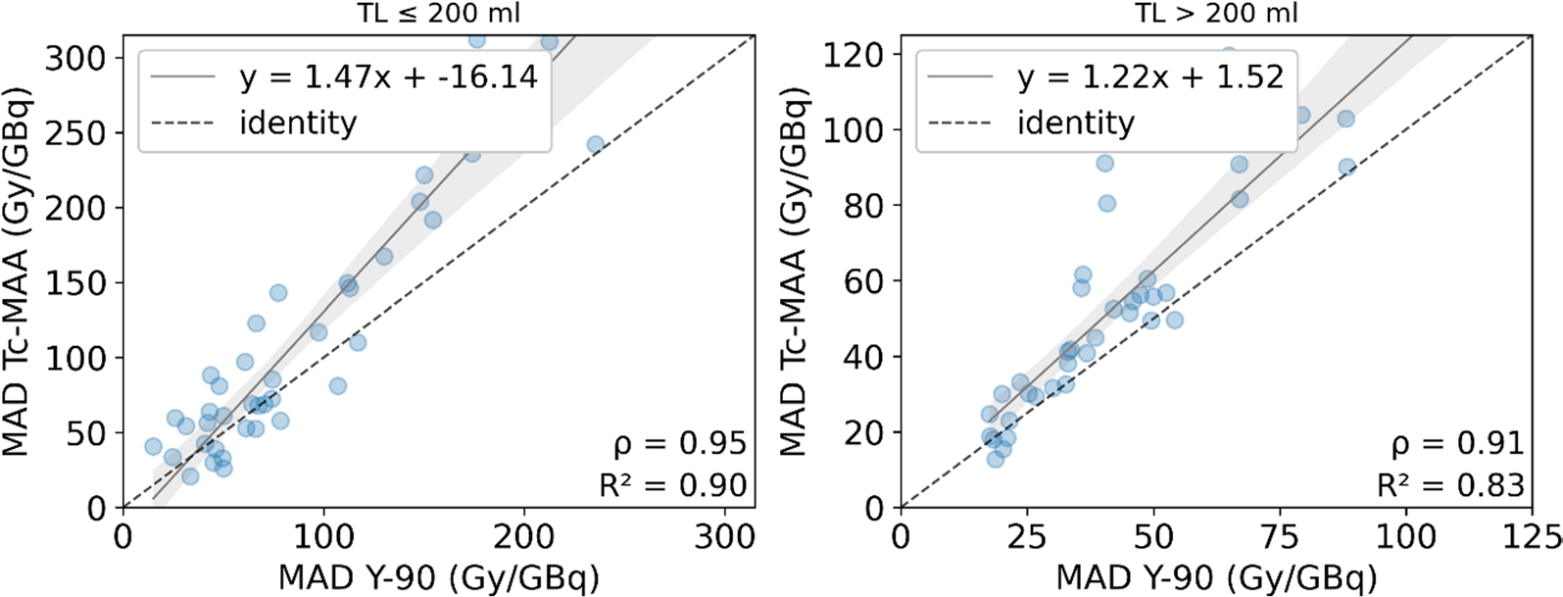
Correlation plots between MAD from simulation (Tc-MAA) and therapy (^90^Y) for TL < 200 ml and TL > 200 ml. For visualization purposes, in each graph the axes are set to equal length, leaving some points out of frame. The entire images are found on Additional file 1: Fig. S4

Tumoral volume was divided into two intervals (TL ≤ 200 ml; TL > 200 ml), and the absorbed dose metrics showing no significant differences in the previous step (namely MAD, D50, D70 and V120) were calculated for each one. The results are shown in Table 3. There is a strong correlation for all the metrics in both groups, although it seems higher for smaller volumes. On the other hand, their relative mean difference is larger, so there is less agreement. Figure 7 shows the correlation plot for both groups.

**Table 3:** Absorbed dose metrics MAD, D50, D70 (Gy/GBq) and V120 (%) compared between simulation (^99m^Tc-MAA) and therapy (^90^Y) for TL divided by tumoral volume

Segment	Metric	^99m^Tc-MAA	^90^Y	Pearson's *r* correlation	Relative difference (%) Mean [range]
**TL ≤ 200 ml** **(n = 25)**	MAD (Gy/GBq)	116.09 ± 101.75	90.23 ± 65.95	0.95 (*P* < .001)	28.27 [− 47.32–170.38]
	D50 (Gy/GBq)	98.36 ± 77.36	85.08 ± 66.31	0.95 (*P* < .001)	19.30 [− 51.72–203.41]
	D70 (Gy/GBq)	79.17 ± 72.02	69.39 ± 54.67	0.95 (*P* < .001)	9.86 [− 58.99–110.75]
	V120 (%)	34.72 ± 25.28	30.16 ± 23.48	0.90 (*P* < .001)	–
**TL > 200 ml** **(n = 39)**	MAD (Gy/GBq)	54.91 ± 31.71	43.84 ± 23.71	0.91 (*P* < .001)	23.70 [− 31.90–125.64]
	D50 (Gy/GBq)	44.06 ± 28.39	39.98 ± 23.09	0.90 (*P* < .001)	8.80 [− 63.17–98.48]
	D70 (Gy/GBq)	28.17 ± 19.50	30.20 ± 18.54	0.86 (*P* < .001)	− 7.00 [− 78.95–78.14]
	V120 (%)	15.88 ± 11.35	12.16 ± 10.88	0.71 (*P* < .001)	–

Wilcoxon’s *p*-value is higher than 0.05 for all metrics

In addition, the difference between the TL absorbed dose metrics between simulation and therapy was calculated for each patient. *P*-value of the Kruskal–Wallis test was not significant (*P* > *0.05*) for all metrics except for D70, which resulted in *P* = *0.04*.

### TNR

**Fig. 8:**
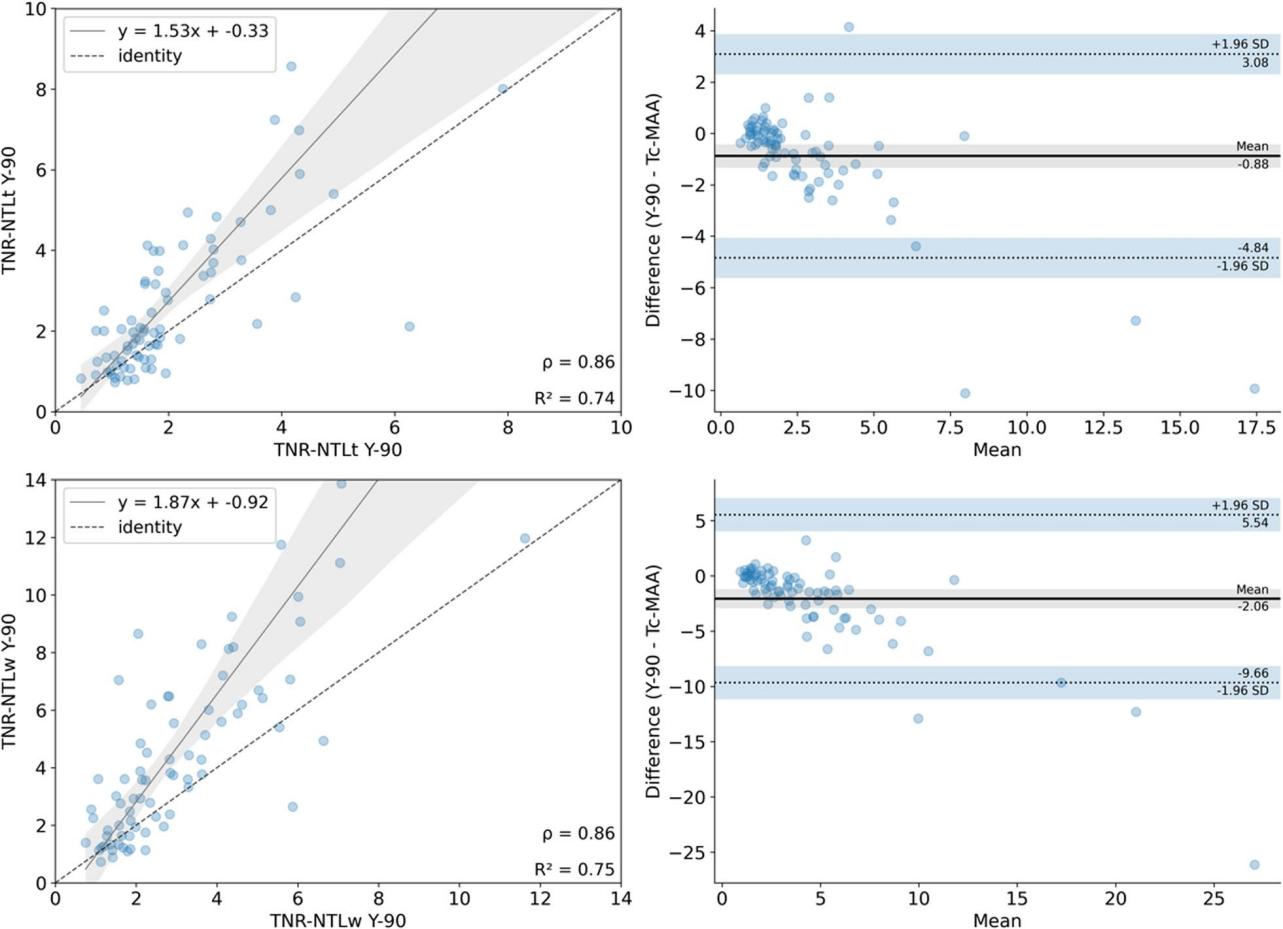
Correlation and Bland–Altman plots for TNR derived from NTLt and NTLw from simulation (top) and post-therapy (bottom)

The TNR was calculated in two ways: by considering the quotient of the activity measured in the TL and (1) NTLt (TNR-NTLt) and (2) NTLw (TNR-NTLw) for both activity maps from SPECTs simulation and therapy (Fig. 8). In addition, the effect of the volume was studied. Results are shown in Table 4. It can be seen that both TNR hold better correlation for TL < 200 ml, whereas larger tumours present significant differences (*P* ≤ 0.05) in both cases.

**Table 4:** Values of TNR (TNR-NTLt and TNR-NTLw) for simulation (^99m^Tc-MAA) and therapy (^90^Y) across the different tumoral liver volumes

Segment	TNR	^99m^Tc-MAA	^90^Y	Wilcoxon *p*-value	Pearson's *r* correlation	Bland–Altman Bias [95% CI]
TL	TL/NTLt	3.14 ± 3.42	2.28 ± 1.94	0.05*	0.86 (*P* < .001)	− 0.86 [− 4.82; 3.09]
	TL/NTLw	5.45 ± 6.00	3.42 ± 2.78	0.01*	0.86 (*P* < .001)	− 2.02 [− 9.61; 5.56]
TL ≤ 200 ml	TL/NTLt	3.54 ± 4.20	2.71 ± 2.43	0.38	0.94 (*P* < .001)	− 0.82 [− 4.87; 3.21]
	TL/NTLw	6.37 ± 7.71	4.11 ± 3.55	0.15	0.90 (*P* < .001)	− 2.26 [− 11.64; 7.11]
TL > 200 ml	TL/NTLt	2.76 ± 2.41	1.83 ± 1.10	0.04*	0.57 (*P* < .001)	− 0.93 [− 4.85; 2.98]
	TL/NTLw	4.58 ± 3.43	2.73 ± 1.41	0.02*	0.67 (*P* < .001)	− 1.85 [− 7.13; 3.43]

^*^ Wilcoxon's *p*-value *P* < 0.05

### Mean absorbed dose difference versus various parameters

A possible correlation between certain parameters and the difference in absorbed dose metrics between simulation and therapy has been evaluated. As parameters of interest, sex, treated lobe and number of lesions were chosen as discrete variables. Age, days between simulation and therapy, LSF and tumour involvement as continuous variables. The Kruskal–Wallis test showed no significant differences for any of the discrete parameters considered, as *P* = 0.80 for sex (masculine/feminine), *P* = 0.17 for treated lobe (right/left) and *P* = 0.57 for number of lesions (single/multiple). In the case of continuous variables, all the analysed parameters showed very weak correlation with the mean difference between ^99m^Tc-MAA and ^90^Y, since *r* = − 0.11 for age, *r* = 0.23 for days between simulation and therapy, *r* = − 0.12 for LSF and *r* = 0.15 for tumour involvement.

## Discussion

The role of personalized dosimetry in SIRT is clear, as it will be crucial in future clinical practice. However, there are still concerns about the use of ^99m^Tc-MAA as a simulation step for therapy. The debate continues as to whether the existing discrepancies between ^99m^Tc-MAA and ^90^Y simulation are related to MAA substitution issues or rather to the ability to administer both compounds under identical conditions [36]. The parameters that may have the greatest influence on a difference in absorbed dose distribution between simulation and therapy are reported to be the number of particles injected, the characteristics of the microspheres (in terms of size, shape, density, material, etc.), differences in catheter position, physiological variations in hepatic blood flow, possible disease progression between pre- and post-images, etc. [19, 22, 31, 38]. However, the quantification of these parameters is challenging.

Therefore, the objective of this study is to establish a practical open-source framework to compare absorbed dose metrics obtained from ^99m^Tc-MAA SPECT images with respect to ^90^Y microsphere theragnostic SPECT images in SIRT in order to develop a suitable workflow for patient selection and personalized absorbed dose planning. We conducted 3D voxel-level dosimetry on a large cohort of 79 patients with 98 index tumours based on self-calibration and local energy deposition method.

According to several studies, there is a strong dose–response correlation with tumoricidal absorbed doses based on simulation with ^99m^Tc-MAA within the range of 205 and 275 Gy [7, 13, 42, 43] and between 160 and 200 Gy for dosimetry based on ^90^Y SPECT/CT or PET/CT [44, 45]. For TL, considering an average about 2.65 ± 1.20 GBq of ^90^Y injected activity, the mean absorbed dose in tumours was estimated as 226 ± 211 Gy and 179 ± 146 Gy, from ^99m^Tc-MAA and ^90^Y, respectively (Table 2), which is in agreement with the previously mentioned literature. The mean absorbed dose to healthy liver (NTLw) was measured in 49.47 ± 22.18 Gy and 54.53 ± 19.78 Gy for simulation and therapy, respectively, and the mean absorbed dose to healthy perfused lobe (NTLt) was measured 86.31 ± 53.98 Gy and 85.54 ± 44.83, respectively. According to the literature, these values are within the limits established for normal tissue impairment [33].

Pearson’s correlation coefficient showed that there is very strong correlation between simulation and therapy mean absorbed doses, either for TL, NTLt and NTLw. Generally, non-tumoral liver doses show stronger correlation than tumoral liver, which may be explained by the higher microsphere heterogeneity and the larger dose gradient within the tumour tissue [33, 41]. Some studies show Pearson *r* correlation coefficients ranging from 0.56 to 0.91 for the tumour simulation-therapy correlation and from 0.71 to 0.99 for non-tumoral liver [16, 17, 19, 23]. The strong correlation found in this study may come from the patient selection criteria (i.e., most of the patients are treated for only one primary lesion, the majority being HCC patients), the exclusion of visually non-matching pre- and post-therapy SPECT images (approximately 11% of the cases) and the normalization of the dose to the total ^90^Y injected activity. However, similarly to previous studies, our results show greater agreement for non-tumoral liver mean absorbed doses (Table 2, Figs. 5, 6).

On the other hand, the absorbed dose values in the tumour calculated with simulation ^99m^Tc-MAA and post-therapy ^90^Y bSPECT were found to be related by a slope greater than unity in the regression curve, suggesting that the former are higher than the latter. The joint histograms (Additional file 1: Fig. S5) also support this trend. They show a pixel-by-pixel correlation between ^99m^Tc-MAA SPECT and ^90^Y SPECT with the fitted line leaning towards ^99m^Tc-MAA (indicated by a slope less than unity in this case). This was presented as a tendency of the simulation to overestimate absorbed doses with respect post-therapy.  A discrepancy between pre-therapy and post-therapy dosimetry has been also reported in both studies using PET [19, 38] and bSPECT [46] as the post-therapy imaging modality. However, as previously discussed, several studies have shown that ^90^Y PET is superior to ^90^Y bSPECT for post-therapy imaging, since the latter presents lower spatial resolution due to the Bremsstrahlung emission. Therefore, it is expected that a discrepancy between predictive and post-therapy dosimetry would be observed when bSPECT is used, which is one of the limitations of using this image modality. However, the main message of our study is showing the strong correlation of the predicted dose from ^99m^Tc-MAA compared to the measured dose from post-therapy ^90^Y bSPECT in terms of microsphere distribution. Our finding is consistent with the results obtained in a phantom study [47] in which the activity of a radioactive source is compared with the activity detected by ^99m^Tc-MAA SPECT, ^90^Y PET and ^90^Y bSPECT. According to their results, the ratio between ^99m^Tc-MAA SPECT and ^90^Y bSPECT activity is 1.6, which is a similar value to the slope obtained in our study, whereas is almost the unity between ^99m^Tc-MAA SPECT and ^90^Y PET. Therefore, it is expected that other studies showing correlation between simulation and therapy with ^90^Y PET will show slopes closer to the unity [17]. Our results in TL agree with another study [46] in which bSPECT is used for post-therapy imaging, presenting the linear correlations for ^90^Y vs. ^99m^Tc-MAA voxel dose from tumours in catheter-matched cases. They obtain a slope of 1.37 with the ^99m^Tc-MAA cases, which is consistent with the slope value obtained in our study for the absorbed dose (Fig. 4).

In addition, we obtained cumulative DVHs generated by voxel dosimetry to compare the heterogeneity of the activity distribution between ^99m^Tc-MAA and ^90^Y. The obtained indices showed good correlation between simulation and therapy. However, significant differences were found for several metrics, mostly on NTL. For TL, D50, D70 and V120 were found to be more correlated, whereas it was V90 for NTL. To the best of our knowledge, there are no studies analysing the agreement and correlation between DVH metrics for glass microspheres on ^90^Y bSPECT.

Our results suggest that the effect of tumoral liver volume does not significantly affect the differences between simulation and therapy for MAD or the rest of DVH metrics. Although the linear correlation was better explained for smaller tumours (TL ≤ 200 ml) than for larger tumours (TL > 200 ml), the latter present less disparity. This is expected and is in agreement with another study [19], as the dosimetry of small lesions is more challenging than larger lesions. They suffer from higher risk of reflux [31], the partial volume effect (PVE) is more severe [31, 33, 48], and segmentation and registration are more complicated, since small variations may lead to large discrepancies on activity quantification [33]. For this reason, lesions smaller than 4 ml (estimated to be 2 cm of diameter) were excluded from the analysis, as recommended by other studies [31, 39].

We have calculated the TNR from simulation and therapy in two different ways, to obtain TNR-NTLt and TNR-NTLw. These results suggest that the TNR obtained from ^90^Y is less variable than from ^99m^Tc-MAA SPECT/CT, either for NTLt and NTLw, which is in agreement with a study performed by Villalobos et al. comparing TNR values from ^99m^Tc-MAA and ^90^Y bSPECT/CT from SIRT with glass microspheres [24]. Both TNR-NTLt and TNR-NTLw in simulation overestimate the TNR in therapy, the latter being statistically lower. This is also consistent with the previous study [24] but contrary to other study by d’Abadie et al. [39], as they found that TNR calculated with ^90^Y imaging is statistically higher than TNR calculated with ^99m^Tc-MAA. Nevertheless, this study uses ^90^Y TOF-PET/CT for post-therapy imaging and is based on SIRT with resin microspheres. The smaller values of TNR found with ^90^Y bSPECT might come from the effect of scattering and PVE.

There is still need to demonstrate feasibility of simulation with ^99m^Tc-MAA, as new methods are emerging to overcome the present discrepancies in the recent years, such as the use of a low-dose ^90^Y scout as a bioidentical surrogate [25] or the use of new radiotracers such as Holmium-166 (^166^Ho)-loaded microspheres as an alternative to ^90^Y-microspheres [49–51]. In addition, although it has been demonstrated that post-treatment dosimetry based on high-resolution PET/CT is superior to bSPECT/CT in terms of image quality [28], SPECT/CT scanners are more available and still widely used in the routine clinical practice [30, 31], so demonstrating the correlation between simulation and bSPECT-based therapy is very valuable in this scenario. There are also some studies showing a significant correlation between absorbed dose metrics calculated from ^90^Y-bSPECT and ^90^Y-PET images [52], paving the way for the use of ^90^Y-bSPECT to establish a robust dose–response relationship. On the other hand, the use of voxel-dosimetry also remains a subject of discussion, as some authors demonstrate its validity in TARE [36], while others [8] show that it does not improve over MIRD approaches.

Due to the lack of a single energy photo-peak in bSPECT, it is common to see wide and varying energy windows employed in different studies for ^90^Y-bSPECT imaging. For instance, Ito et al. [53] compared several energy windows and concluded that the 102–138 keV window provided the highest resolution and lowest uncertainty, Rhong et al. [54] found 80–180 keV to be the optimal window, and Roshan et al. [55] preferred 60–400 keV. In addition, another study comparing different window settings, similar to those used in this study, showed equivalent signal-to-background ratios [56]. In this study, patients were imaged after therapy with three different energy window settings, which may affect image quality. However, in a prior analysis, we verified that the correlations between simulation and therapy did not depend on the choice of window. Therefore, no distinction was made between window settings in this study.

Our findings support other studies claiming good agreement between simulation and therapy; however, there are several limitations. The reconstruction of SPECT images did not include scatter correction, which may influence the results, because as suggested by other studies, this may lead to an overestimation of up to 40% in the absorbed dose for non-tumoral tissue [57]. However, it represents real-life clinical practice, where these corrections are not always available. It should also be noted that the use of bSPECT instead of PET as a post-therapy imaging method exacerbates the discrepancies between simulation and therapy due to its poorer spatial resolution, as previously discussed. Cases that did not visually match simulation and therapy were excluded from the mean calculations. This selection of patients was done with the aim of eliminating the physical differences between simulation and therapy as a source of uncertainty, as a voxel-to-voxel analysis is expected to be different in these cases (Fig. 2), and to investigate possible sources of discrepancy in other factors. Nevertheless, it would be prudent to perform a separate analysis including these cases, as they represent approximately 11% of the total number of cases, a non-negligible percentage of patients, and are again representative of real-life scenarios. In addition, motion correction due to breathing was not applied for lesions dwelling in the superior hepatic lobe, which can cause a mismatch between CT and SPECT at the dome of the liver and attenuation correction issues [31].

Images were registered using rigid transformations, as recommended by other authors [31, 58], since deformable registration can cause differences in matrix and voxel size. On the other hand, rigid registration does not take into account changes in the geometry of the abdominal organs, which may result in missing counts when calculating activity and aggravate co-registration errors. In addition, no information was available on the time delay between ^99m^Tc-MAA administration and ^99m^Tc-MAA SPECT imaging. However, it would be worth investigating the effect of this factor on the predictive ability, as particle degradation may also become a source of uncertainty. As future work, it is intended to perform the same analysis but applying the previous corrections. Also, it would be interesting to study the behaviour of the excluded necrotic areas.

In this study, it has been shown that there is a strong correlation between the absorbed dose metrics extracted from ^99m^Tc-MAA simulation SPECT/CT images and the post-therapy images with ^90^Y SPECT/CT based on voxel dosimetry, highlighting the predictive ability of ^99m^Tc-MAA. In addition, different absorbed dose parameters derived from DVHs, representing the microspheres distribution, have been evaluated, confirming the strong correlation between simulation and therapy absorbed dose distribution.

## Conclusion

The simulation step with ^99m^Tc-MAA SPECT/CT in SIRT plays an important role in the selection of potential candidates to therapy as a theragnostic approach for treatment planning. Despite controversy about its usefulness, it has been found that there is a high correlation between mean absorbed doses on tumoral and non-tumoral liver, even with post-therapy bSPECT imaging, highlighting the predictive value of dosimetry based on ^99m^Tc-MAA SPECT. In addition, DVH metrics provide important information on the heterogeneity of dose distribution within segments; hence, voxel-dosimetry is highly supported.

## Supplementary Information

**Additional file 1**. Additional information regarding the absorbed dose calculation method. Figs. S1–S4 show the same correlation plots as Figs. 4–7 but without trimming the axes, so the unit line does not necessarily go from the origin to the upper right corner. Fig. S5 shows the joint histograms for simulation and therapy from NTLw, NTLt and tumours.

## Data Availability

The dataset analyzed during the current study is not publicly available and cannot be shared due to ethical standards.

## Abbreviations

^90^Y: Yttrium-90
^99m^Tc: Tecnecium-99 metastable
^99m^Tc-MAA: 99MTc-macroaggregated albumin
^166^Ho: Holmium-166
AC: Attenuation-corrected
bSPECT: Bremsstrahlung SPECT
CT: Computed tomography
DVH: Dose volume histogram
EBRT: External beam radiation therapy
HCC: Hepatocellular carcinoma
ICC: Intrahepatic cholangiocarcinoma
LSF: Lung shunt fraction
MAD: Mean absorbed dose
mCRC: Metastatic colorectal cancer
MR: Magnetic resonance
NTL: Non-tumoral liver
NTLt: Non-tumoral liver target
NTLw: Non-tumoral whole liver
OSEM3D: Three-dimensional ordered-subset expectation maximization
PET: Positron emission tomography
PVE: Partial volume effect
RE: Radioembolization
REILD: Radioembolization induced liver disease
SD: Standard deviation
SIRT: Selective internal radiation therapy
SPECT: Single-photon emission computed tomography
TL: Tumoral liver
TNR: Tumour-to-normal liver ratio
TOF: Time-of-flight
VOI: Volume of interest
