# Supplementary material for: Predictive value of 99mTc-MAA-based dosimetry in personalized 90Y-SIRT planning for liver malignancies
Source: EJNMMI Res. 2023 Jul 3;13:63. doi: 10.1186/s13550-023-01011-3 (PMC10317941; doi:10.1186/s13550-023-01011-3)
Supplement: Supplementary file 1 — Additional file 1. Additional information regarding the absorbed dose calculation method. Figs. S1–S4 show the same correlation plots as Figs. 4–7 but without trimming the axes, so the unit line does not necessarily go from the origin to the upper right corner. Fig. S5 shows the joint histograms for simulation and therapy from NTLw, NTLt and tumours. [file 13550_2023_1011_MOESM1_ESM.pdf]

Predictive Value of ^{99m}Tc-MAA-based Dosimetry in Personalized ^{90}Y-SIRT planning for Liver Malignancies

Mercedes Riveira-Martin[1], Azadeh Akhavanallaf[2], Zahra Mansouri[2], Nicola Bianchetto Wolf[2], Yazdan Salimi[2], Alexis Ricoeur[3], Ismini Mainta[2], Valentina Garibotto[2,4,5], Antonio López Medina[6*], Habib Zaidi[2,5,7,8†]

[1] Galicia Sur Health Research Institute, Department of Medical Physics and RP, Vigo, Spain

[2] Division of Nuclear Medicine and Molecular Imaging, Diagnostic Department, Geneva University Hospital, Geneva, Switzerland

[3] Service of Radiology, Geneva University Hospital, CH-1211, Geneva, Switzerland

[4] Center for Biomedical Imaging (CIBM), Geneva, Switzerland

[5] Geneva Neuroscience Center, Geneva University, Geneva, Switzerland

[6] Hospital do Meixoeiro (GALARIA), Department of Medical Physics and RP, Vigo, Spain

[7] Department of Nuclear Medicine and Molecular Imaging, University of Groningen, University Medical Center Groningen, Groningen, Netherlands

[8] Department of Nuclear Medicine, University of Southern Denmark, Odense, Denmark

Corresponding authors

Antonio López Medina, Ph.D*
Hospital do Meixoeiro (GALARIA)
Department of Medical Physics and RP, Vigo, Spain
Tel: +34 986810921
email: antonio.lopez.medina@sergas.es

Habib Zaidi, Ph.D†
Geneva University Hospital
Division of Nuclear Medicine and Molecular Imaging, CH-1211 Geneva, Switzerland
Tel: +41 22 372 7258
Fax: +41 22 372 7169
email: habib.zaidi@hcuge.ch

Supplemental:

Absorbed dose calculation:

The dosimetry calculations were performed using a self-developed MATLAB code (MATLAB (2021a), Natick, Massachusetts: The MathWorks Inc) according to the following pipeline, based on the calculations described by Moran et al. [40]:

a) 3D-voxel activity maps are calculated for both ^{99m}Tc-MAA and ^{90}Y SPECTs using a self-calibration factor (administered activity/total counts within the liver in the SPECT image) [26, 40-42]. The initial activity in each voxel comes as:

$$A_{vox}(x) = \frac{C_{vox}(x)(1 - \mathrm{LSF} - \mathrm{Res})\mathrm{A}^{90\mathrm{Y}}}{C_{WL}(x)} \quad (1)$$

Where x can be ^{90}Y or ^{99m}Tc-MAA calibrated images, Res is the residual activity and WL refers to the whole-liver segment.

b) The total number of disintegrations in a voxel can be calculated under (1) as:

$$\widetilde{\mathrm{A}}_{\mathrm{vox}}(x) = 1.443 \cdot T_{1/2}(^{90}Y) \cdot A_{vox}(x) \quad (2)$$

c) 3D-voxel absorbed dose maps are calculated based on the LDM. Since this model presumes that the kinetic energy of every beta emission is deposited within the voxel where it occurs (target = source), the absorbed dose in each voxel is calculated by multiplying the cumulative activity within the voxel ($\widetilde{\mathrm{A}}_{\mathrm{vox}}$) by a constant scalar factor (S value).

$$D_{\mathrm{vox_t}}(x) = \tilde{A}_{\mathrm{vox_s}}(x) \times S(\mathrm{vox}_t \leftarrow \mathrm{vox}_s)\big|_{t=s} \quad (3)$$

The S value can be calculated as:

$$S(\mathrm{vox}_t \leftarrow \mathrm{vox}_s)|_{t=s} = \left.\frac{\langle E_\beta(^{90}Y)\rangle}{M_{\mathrm{vox}_t}}\right|_{t=s} \quad (4)$$

M_{vox} stands for the mass of the voxel and $\langle E_\beta(^{90}Y)\rangle$ for the averaged beta energy deposited per disintegration, which is calculated under (4).

d) Simplifying the equations, the 3D-voxel dose maps can be calculated as:

$$D_{\mathrm{vox}}(x) = 2.14 \cdot 10^{-4} \cdot T_{1/2}(^{90}Y) \cdot A_{vox}(x) \quad (5)$$

Where D_{vox} is expressed in Gray (Gy), $T_{1/2}(^{90}Y)$ in seconds (s) and A_{vox} in giga-becquerel (GBq).

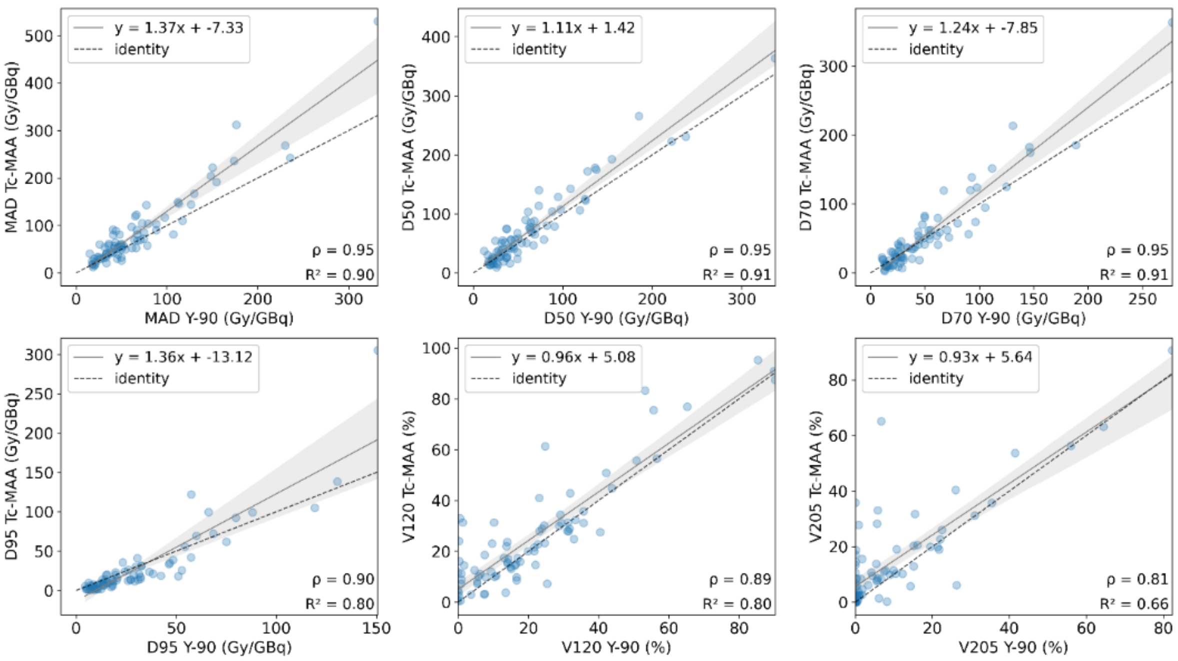

Fig. S1 Correlations plots for different dosimetry metrics derived from TL: mean dose (MAD), D50 and D70 (top) and D95, V120 and V205 (bottom). All points are shown

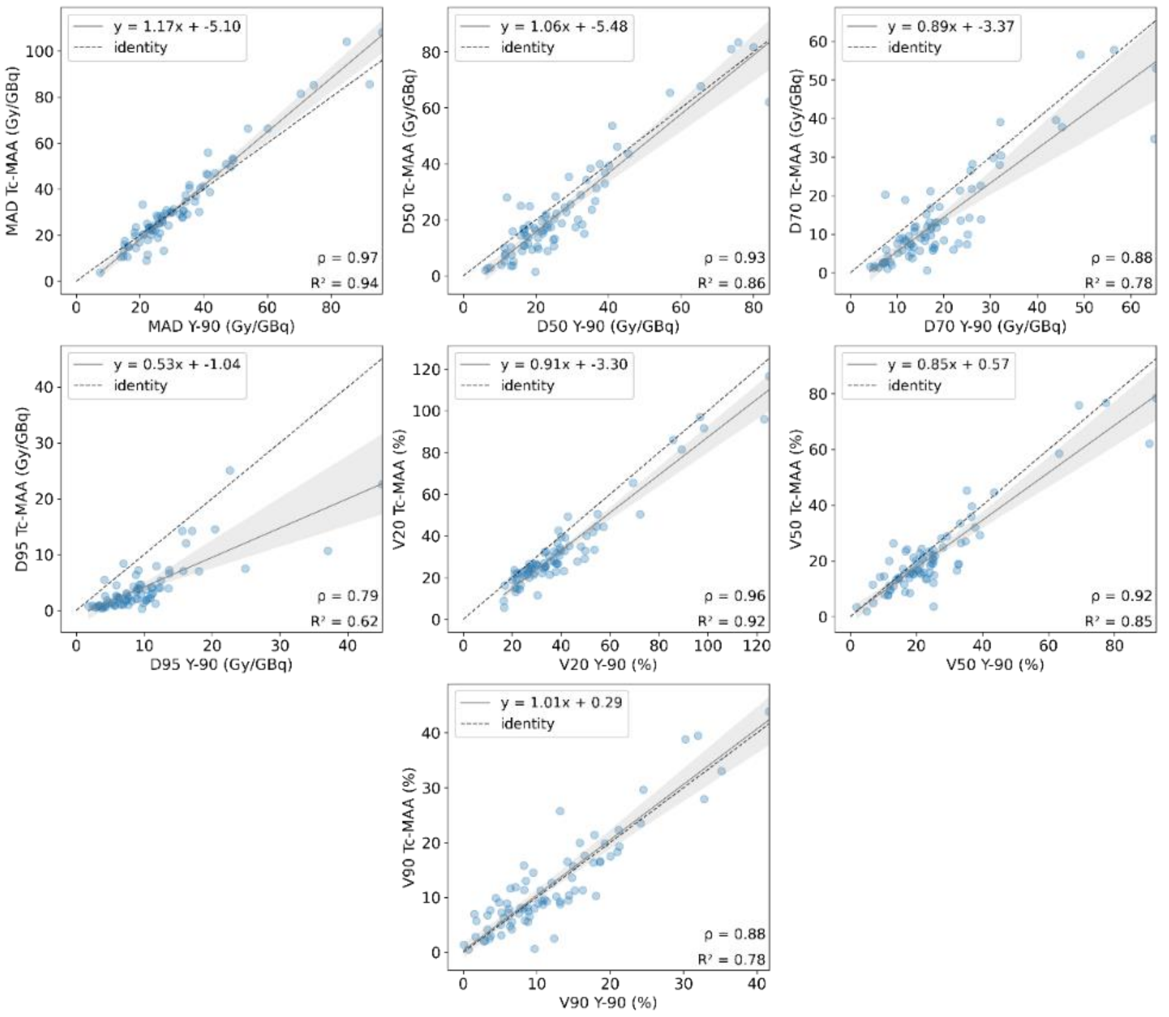

Fig. S2 Correlations plots for different dosimetry metrics derived from NTLt: mean dose (MAD), D50 and D70 (top) and D95, V50 and V90 (bottom). All points shown

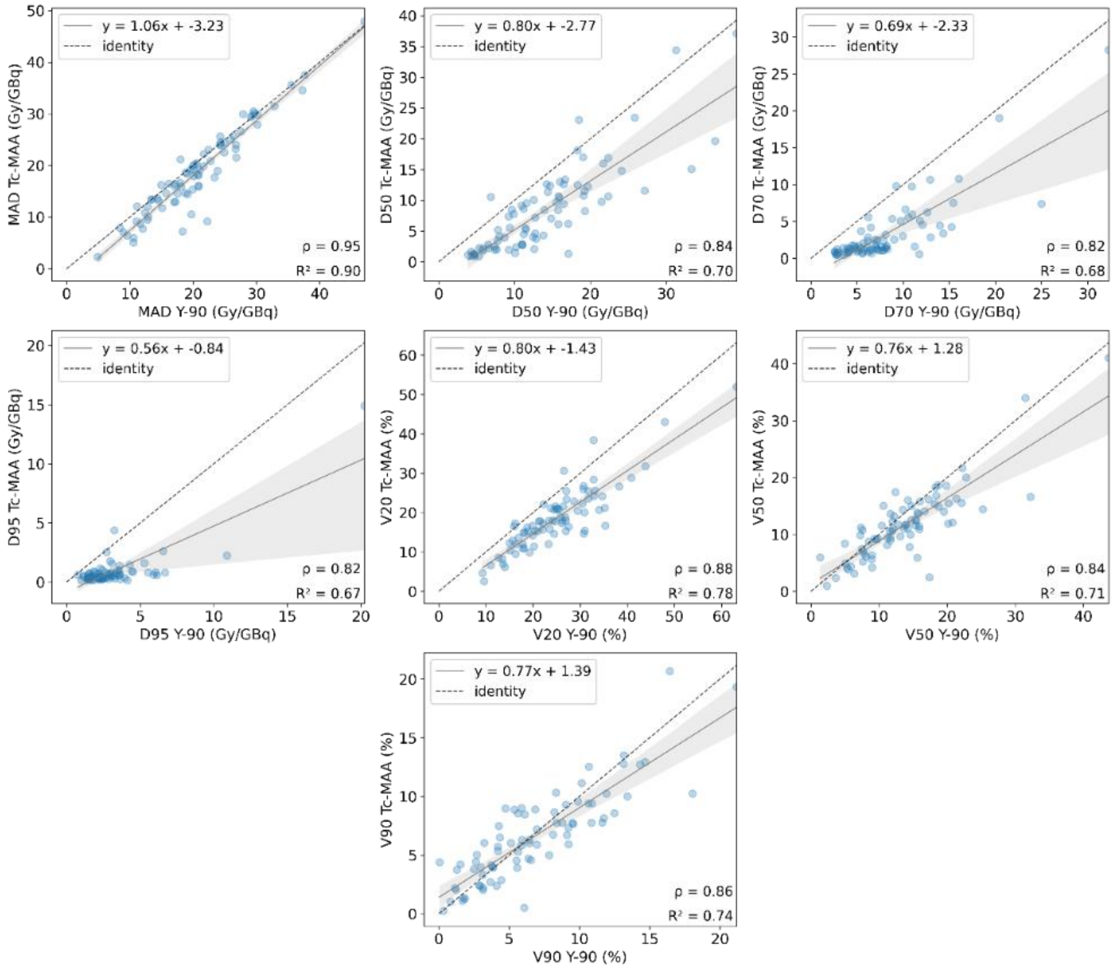

Fig. S3 Correlations plots for different dosimetry metrics derived from NTLw: mean dose (MAD), D50 and D70 (top) and D95, V50 and V90 (bottom). All points shown

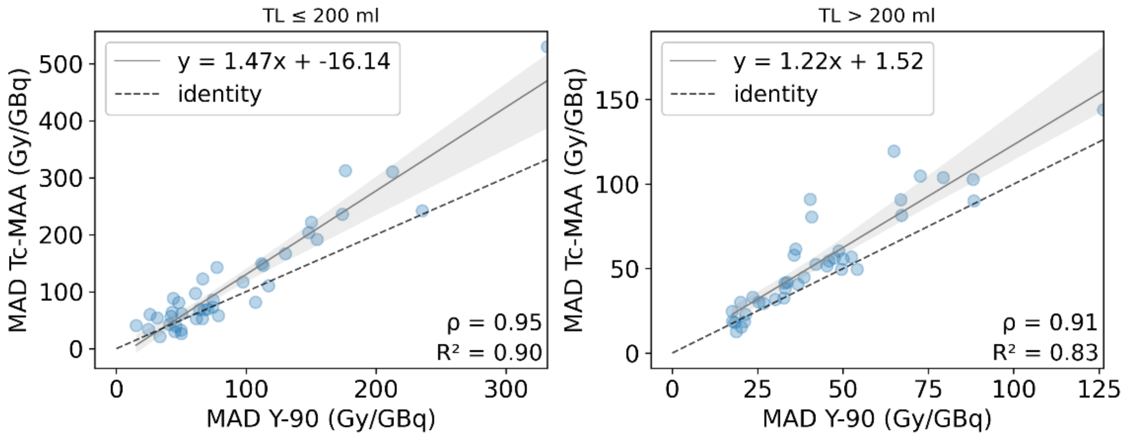

Fig. S4 Correlation plots between MAD from simulation (Tc-MAA) and therapy (90Y) for TL < 200 ml and TL > 200 ml

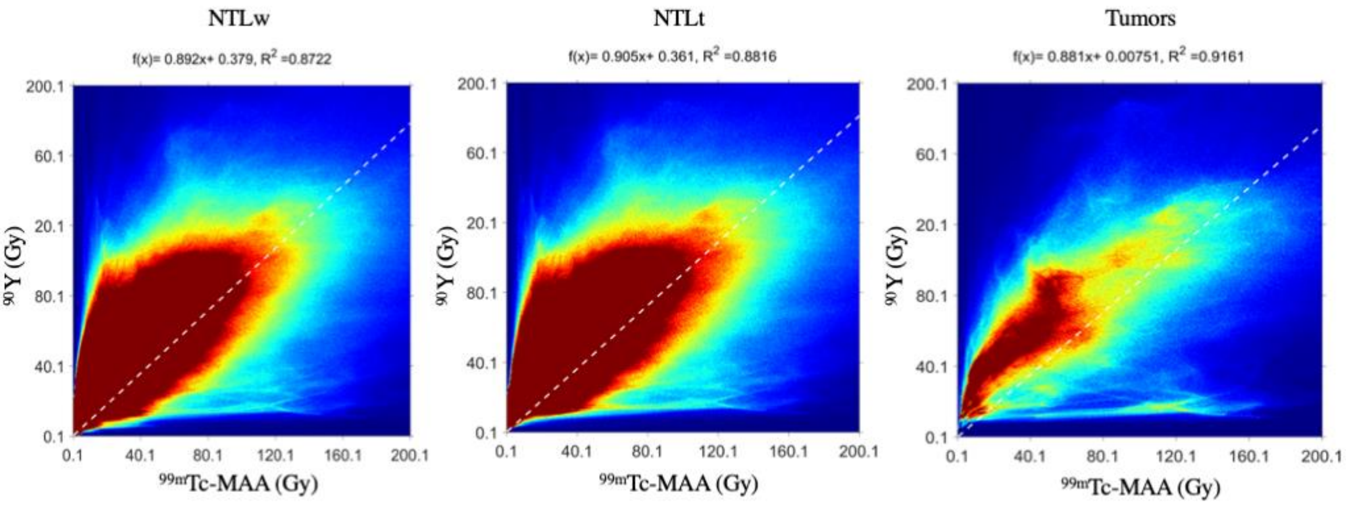

Fig. S5 Joint histograms for simulation and therapy from NTLw, NTLt and tumors. The fitted line is showed as a dashed line, while the regression model is shown on top of each graph
